# Scarlet Fever in Iran During the Qajar Period (1796 to 1925 AD); A Brief Historical Review

**DOI:** 10.34172/aim.35069

**Published:** 2025-11-01

**Authors:** Seyyed Alireza Golshani, Ghobad Mansourbakht, Mehrdad Haghighi

**Affiliations:** ^1^Department of History, Shahid Beheshti University, Tehran, Iran; ^2^Department of Infectious Disease, Imam Hossein Educational Hospital, School of Medicine, Shahid Beheshti University of Medical Sciences, Tehran, Iran

**Keywords:** 19th Century histories, History of medicine, Iran, Pandemics, Scarlet fever

## Abstract

Scarlet fever, known in Persian as "*Tab-e Sorkh*," is a bacterial infection caused by *Streptococcus pyogenes*. During the Qajar era (1796–1925), it was often deadly and reached pandemic levels in the 19th century. Both traditional Iranian and European medicine described its symptoms, but few comparative studies exist. By analyzing historical Persian texts, European medical reports, and modern literature, this study compares how the disease was understood and treated. Symptoms like rash and fever were widely recognized, and mortality was high before antibiotics. Traditional treatments followed humoral theory, including herbal remedies and bloodletting. Outbreaks peaked in cold months, mostly affecting children aged 5–15 years — a pattern seen in both medical systems. From the 1820s to 1880s, scarlet fever caused global outbreaks, especially in Iran. This research shows how combining historical perspectives can deepen our understanding of infectious diseases and their treatment across time.

## Introduction

 Scarlet fever, a toxin-mediated complication of *Streptococcus pyogenes* (Group A Streptococcus) infection, is one of the most significant infectious diseases in medical history. It arises from a delayed-type hypersensitivity reaction to erythrogenic (pyrogenic) exotoxins, predominantly types A, B, and C, with type A responsible for the majority of cases and types B and C accounting for the remainder. Clinically, the disease is characterized by fever, pharyngitis, a diffuse erythematous “sandpaper” rash, and a strawberry tongue, and was historically a leading cause of morbidity and mortality among children.^1,2^

 Beyond its medical implications, scarlet fever holds historical and social importance. Examining its evolution over time, how societies responded to it, and its impact on healthcare systems provides valuable insight into humanity’s interaction with infectious diseases.^3^ This article begins by reviewing authoritative scientific literature to trace the history of scarlet fever from its earliest clinical descriptions to its control in the modern era.

 The earliest historical accounts of scarlet fever can be examined as follows: while the disease is not explicitly described in ancient texts as it is understood today, some medical historians believe that its symptoms may be observed in the writings of Hippocrates (460–370 BC) and Galen (129–216 AD).^4^ However, the 17th-century British physician, Thomas Sydenham (1624–1689), is recognized as the one who provided the first precise description of scarlet fever. In 1676, Sydenham introduced the term *febris scarlatina* and distinguished the disease from other febrile illnesses such as measles and smallpox.^5^

**Figure 1:**
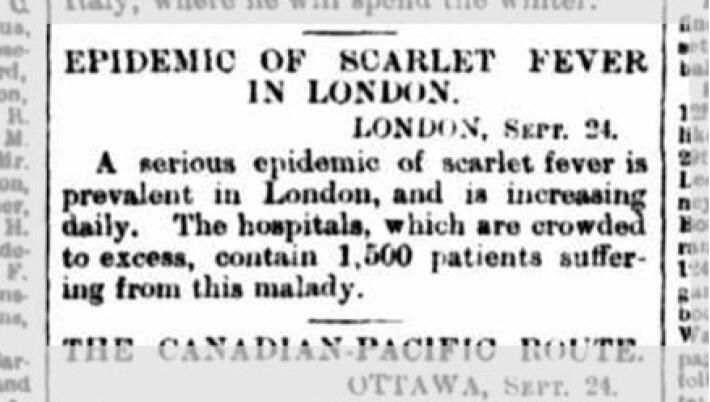
Epidemic of Scarlet Fever In London. London, Sept. 24. (Published Monday, Sept. 26, 1887). A serious epidemic of scarlet fever is prevalent in London, and is increasing daily. The hospitals, which are crowded to excess, contain 1,500 patients suffering from this malady^8^

**Figure 2:**
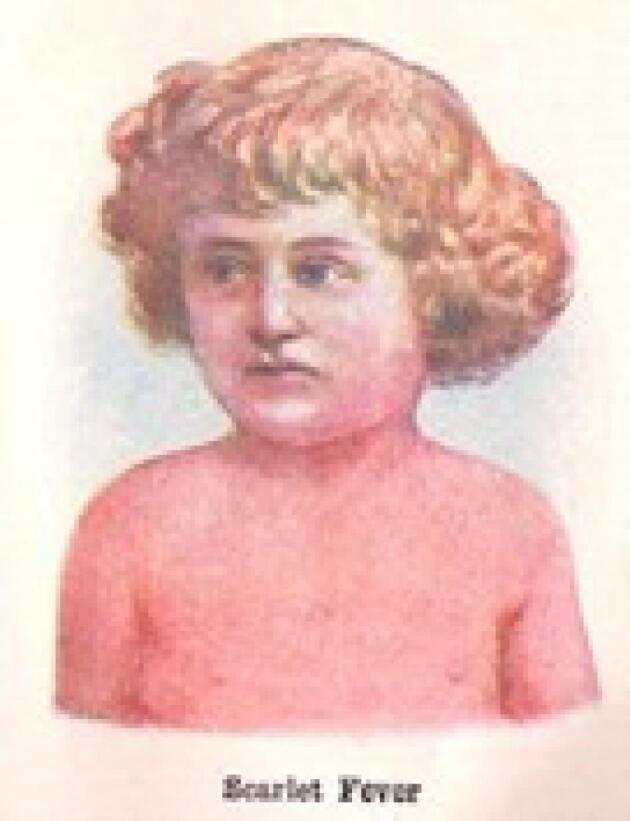
Clinical Illustration from 1908 Depicting the Characteristic Rash of Scarlet Fever. The original image was part of a diagnostic comparison between scarlet fever and measles, reflecting early 20th-century efforts to differentiate exanthematous diseases based on rash morphology^9^

 Between 1825 and 1885, scarlet fever became a prevalent disease in Europe, causing repeated epidemics. During this period, limited knowledge about its bacterial origin and lack of effective treatments led to high mortality rates, particularly among children.^6^ The 19th century witnessed severe outbreaks of scarlet fever across the globe, transforming it into one of the leading causes of child mortality. Historical records from Britain reveal that during the 1840s, the disease claimed thousands of lives annually. Similarly, widespread outbreaks in the United States posed significant challenges to public health systems^7^ (Figures 1 and 2).

**Figure 3:**
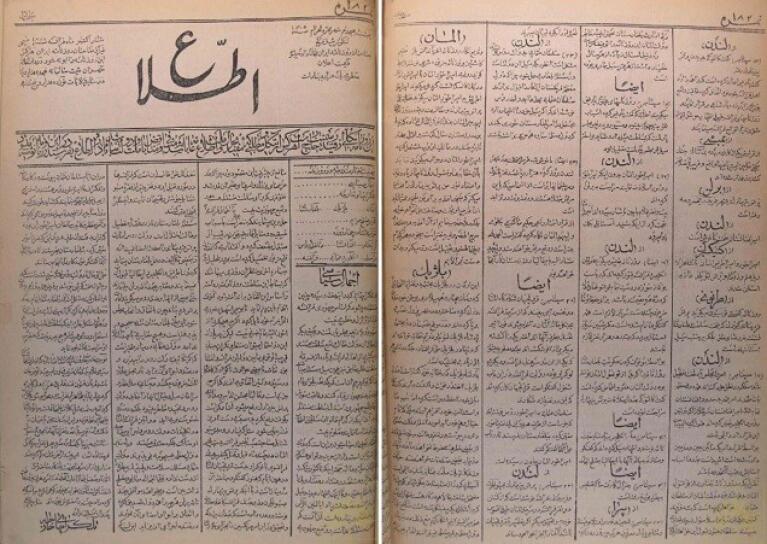
Outbreak of Scarlet Fever in London as Reported in *Ettela* Newspaper, Issue 182, October 5, 1887^10^

 The 1887 outbreak of scarlet fever in London appears to have been significant enough to be reported in Qajar-era Persian publications. The newspaper *Ettela*, in its 182nd issue, reported on the severe outbreak in London as follows: “The red fever illness (scarlet fever) has appeared in the city of London, with fifteen hundred people sick and hospitals filled with those afflicted by this disease, increasing day by day”.^10^ While the reported casualty figures were high, the article notably omitted any details regarding symptoms or treatment methods (Figure 3).

 One of the primary reasons for the high mortality rate of scarlet fever during this period was the poor living conditions in industrial cities. Overcrowding, inadequate sanitation, and malnutrition served as predisposing factors for the spread of streptococcal infections.^11^

 By the late 19th century, significant progress had been made in understanding the etiology of scarlet fever. In 1883, the German bacteriologist Friedrich Johann Loeffler (1852–1915) successfully isolated streptococci from scarlet fever patients, though he was unable to definitively establish the causal relationship between the bacterium and the disease.^12^ It was not until the 1920s that the work of Gladys Rowena Henry Dick (1881–1963) and George Frederick Dick (1881–1967) confirmed *Streptococcus pyogenes* as the primary causative agent of scarlet fever, with its erythrogenic toxin responsible for the characteristic skin rash.^13^

 The 1928 discovery of penicillin by Sir Alexander Fleming (1881–1955), a Scottish microbiologist and physician, and its subsequent widespread use in the 1940s, revolutionized the treatment of Scarlet Fever. Antibiotics not only reduced the severity of the disease but also prevented dangerous complications such as rheumatic fever and glomerulonephritis.^14^

 Despite a dramatic decline in scarlet fever cases during the 20th century, recent reports indicate a resurgence of the disease in certain regions, including the UK and East Asia. Studies suggest that the emergence of new Group A Streptococcus strains capable of producing more potent toxins may be contributing to this increase.^15^

 In the 19th century, scarlet fever was particularly prevalent in densely populated areas with poor sanitation, such as cities in the Ottoman Empire (1299–1923)^16^ and Russian Empire (1721–1917).^17^ Given that epidemics of contagious diseases like the plague,^18^ cholera,^19^ Russian flu,^20^ Spanish flu^21-25^), typhus,^26,27^ typhoid fever, smallpox, pneumonia and measles,^28^ entered Qajar Iran from Tsarist Russia and the Ottoman Empire, it is plausible that scarlet fever may have followed a similar route. During this period, military, commercial, and diplomatic interactions between Tsarist Russia, the Ottoman Empire, and Qajar Iran intensified. The frequent movement of soldiers, merchants, and travelers between these regions could have facilitated the transmission of scarlet fever. However, despite extensive research, no direct historical records documenting its presence in Iran during the Qajar era have been found. The history of scarlet fever exemplifies the complex interplay between humans and infectious diseases. From its obscure early descriptions in ancient texts to the identification of its bacterial cause and the advent of antibiotic treatment, this disease has had a profound impact on human societies. Today, despite medical advancements, ongoing surveillance of scarlet fever epidemiology and research into resistant bacterial strains remain crucial.

 This study marks the first comprehensive investigation of scarlet fever (both as an epidemic and seasonal disease) during the Qajar period (1796–1925), examining contemporary perspectives from modern physicians, traditional healers, historians, and journalists.

 The central question this research seeks to address is the high prevalence of scarlet fever among children aged 5 to 15 in Qajar Iran (1796–1925), a topic that has not been sufficiently explored in previous studies. This research aims to adopt a narrative and historiographical approach to analyze the causes and factors behind the spread of scarlet fever in Iran, as well as the measures taken to combat it and the treatment methods employed. Finally, the study examines how this disease was reflected in medical texts, historical sources and Qajar-era newspapers.

## Scarlet Fever Before the Qajar Period and in Iranian Medical Texts

 Scarlet fever, known in Persian by various terms including “*Tab-e Sorkh*”, “*Marz -e Mokhmali*”, “*Mokhmalak*”, “*Shari*”, “*Gol- Afshan*” and “*Margejeh*” (Nettle rush; urticaria),^29^ was documented by several prominent Persian physicians. Scholars such as Rhazes, Avicenna, Haly Abbas, and Jurjani made references in their works to a disease resembling scarlet fever, describing it as similar to measles but with a lighter rash and more severe course. However, the precise correlation between these historical descriptions and actual scarlet fever cases remains uncertain.^4^

 The most definitive historical reference to scarlet fever in Iran appears in the account of Georg Tectander von der Jabel, the German author of Iter Persicum. Little is known about Tectander’s life except that he served as secretary to István Kakas de Zalonkemeny, a Transylvanian noble and ambassador from Rudolf II (1552‒1612) of the Habsburg dynasty, Holy Roman Emperor, to the court of Shah Abbas the Great (r. 1588‒1629). According to Tectander’s narrative, the German embassy arrived in Moscow in August 1602, with Kakas subsequently traveling on a Russian ship across the Caspian Sea to Langarud in Iran. Tragically, most delegation members, including Kakas himself, succumbed to scarlet fever in Iran. On his deathbed, Kakas entrusted Tectander with delivering the Holy Roman Emperor’s letter to Shah Abbas. Even Kakas’s successor contracted the disease, though he survived.^30^

 This outbreak likely originated in Moscow (a region historically endemic for scarlet fever) with the disease traveling with the diplomatic party to Iran. The cold climate of Moscow, combined with the seasonal patterns of streptococcal infections, may have facilitated this transmission. This account represents the earliest documented epidemic of scarlet fever in Iranian history, predating by two centuries the disease’s recognition in European medical literature. The severe impact on the diplomatic mission underscores both the virulence of this particular strain and the absence of immunity among the travelers.

## Scarlet Fever in Qajar Iran: Diagnostic Challenges and Medical Perspectives

 During the Qajar period, scarlet fever represented a significant infectious disease burden, presenting considerable diagnostic and therapeutic difficulties for physicians. Contemporary medical observers offered conflicting accounts regarding its epidemiology in Persia. James M. Barker, an English physician practicing in Iran, postulated the disease’s introduction around 1855. In contrast, Dr. Johan Louis Schlimmer (1819‒1881), the Dutch physician and instructor at Tehran’s Dar al-Funun medical school, maintained (based on his clinical experience and consultations with local practitioners) that the disease had existed endemically prior to this date.^31,32^ Schlimmer provided particularly insightful critiques of his European colleagues’ diagnostic limitations. He noted how physicians like Dr. Jakob Eduard Polak (1818‒1891) and Hantzsche, German physician, frequently denied scarlet fever’s presence in Persia and the broader Eastern regions, primarily due to their restricted opportunities to observe early-stage presentations. This epistemological gap led Schlimmer to advocate for more substantive collaboration between European and Persian physicians, urging foreign doctors to attend local clinics to better understand indigenous diagnostic frameworks and therapeutic approaches.^33^

 Persian physicians in the Khorasan province had developed clinically meaningful distinctions between: Mokhmalak”, “Shari”, “Gol-Afshan” and “Margejeh”:

1. Benign scarlet fever (*Mokhmalak*)
2. Malignant scarlet fever (*Margejeh*): considered invariably fatal, which Schlimmer hypothesized might represent gangrenous or septicemic variants.^33^

Dr. Polak’s clinical records reveal fascinating parallels, documenting several exanthematous conditions with scarlet fever-like presentations:

- "Buthorat-e Gazneh-i” (nettle-type rashes)
- “Kahir” (urticarial eruptions)
- “Anjuriyeh” and “Nabat al-Lail”: autumnal febrile exanthems with malarial characteristics
- Notably observing their particularly severe manifestation among non-acclimated travelers.^34^

During the Qajar era, scarlet fever, primarily perceived as a dermatological condition, was known to spread through contact with infected individuals or contaminated objects. While generally unnamed across most Persian provinces, the disease carried the distinctive appellation “Mokhmalak” (scarlet fever) in Kerman’s regional lexicon.^34^ The infection manifested with grave systemic complications including renal involvement, severe otitis media, lymphadenopathy, endocardial damage, and life-threatening septicemia.

 The eminent Dutch historian and Iranologist Willem Marius Floor posits compelling evidence of the disease’s pre-Qajar presence in Persia, noting two significant indicators: Armenian women’s specialized therapeutic knowledge and Persian physicians’ clinical distinction between malignant and benign variants.^35^ Epidemiologically, the disease demonstrated marked seasonality, with winter months bringing heightened incidence among pediatric populations, though adult cases were not uncommon.

 Traditional treatment protocols centered on hydrotherapy, with families transporting patients to mineral spring regions such as Shemiran’s renowned thermal waters. Floor dates Persia’s formal medical recognition of scarlet fever to approximately 1855.^35^ Endemic zones primarily included the Khamseh province (Qazvin-Tabriz corridor), Qaradagh, and Khalkhal regions, where the disease’s social perception bore striking parallels to leprosy, both in presumed heredity transmission and ritual impurity ascribed to sufferers.^34^ This stigmatization precipitated severe quarantine measures, barring infected individuals from urban centers and confining them to specialized leper colonies in Azerbaijan and Khorasan.^35^

 The clinical presentation followed a characteristic progression:

- Initial eruption of fine papules between eyebrows
- Desquamation of lesional skin
- Advanced manifestations: alopecia, nasal ulceration, oropharyngeal mucosal involvement
- Terminal phase: eyelid deformation and osteomyelitic complications.^34^

His accounts further describe:

- Pemphigus contagiosus in pediatric populations (often fatal in infants)
- Amebic dysentery exhibiting distinct seasonal patterns (August to mid-November).^34^

The clinical descriptions provide invaluable paleoepidemiological data, while the professional debates underscore the colonial-era power dynamics in medical knowledge production. Particularly noteworthy is Schlimmer’s exceptional approach, combining rigorous European diagnostics with respectful engagement of Persian medical wisdom, representing a rare model of cross-cultural medical practice in the 19th century colonial medicine.

## Response of Qajar-Era Historians and Journalists to Scarlet Fever

 In addition to European physicians who documented scarlet fever in their memoirs and travelogues, Iranian historians, journalists, and memoir writers also made numerous references to this disease. Even in the press of the Nasiri period, there are brief mentions of the disease. However, the limited information reflected in newspapers makes it difficult to form even a general opinion about the spread of scarlet fever in Iran. This is because no reports have explicitly described the disease as epidemic in nature. The only exception appears in the pages of the newspaper “Iran”, where, during a discussion about the outbreak of sore throat illness, mention is made of an outbreak of scarlet fever in the city of Isfahan. Although our data on this disease is limited to just this one news report, it contains valuable information. First, the date of this report roughly coincides with the time when Schlimmer spoke of the disease’s spread in Iran, thus serving as confirmation of his observations. Second, the article includes descriptions of symptoms such as fever, sore throat, and rashes. Another important detail is that the outbreak was reported to have occurred in autumn, consistent with what we know today: scarlet fever tends to be more common in colder seasons.^36^

**Figure 4:**
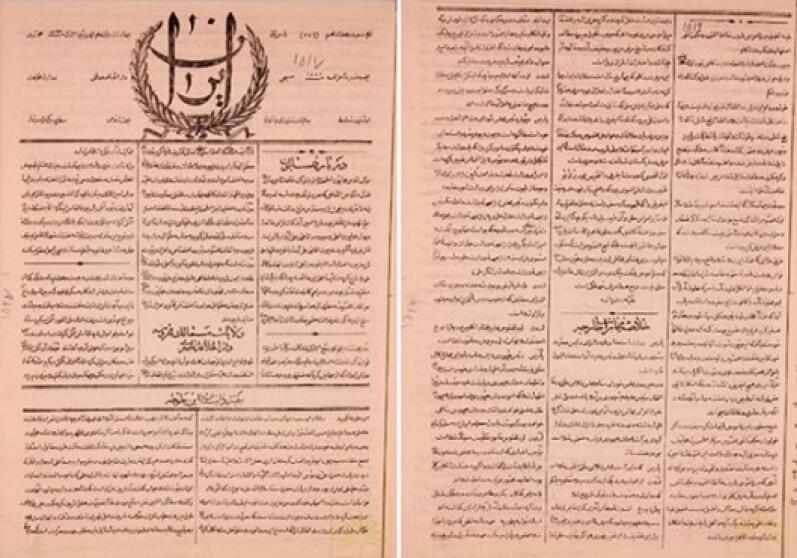
Newspaper Iran, Issue No. 379, dated March 5, 1879^37^

 The newspaper Iran described the outbreak in Isfahan as follows: “…Six years ago (1290 AH / 1873), scarlet fever (*Mokhmalak*) appeared in Isfahan in two waves, manifesting in its usual and regular manner. Its symptoms included fever, mild sore throat, small red spots all over the body, and in some cases, smooth, bright red patches covering the entire skin surface, eventually leading to peeling of the skin like bran. The first wave occurred in autumn and was highly prevalent and severe, lasting for about two months. In the second year, initially, a widespread outbreak of smallpox occurred, followed later by a more moderate but still widespread occurrence of scarlet fever...”.^37^ This report aligns with Schlimmer’s observations, confirming that the disease tended to spread in autumn (Figure 4).

 Description in the Newspaper Iran: The newspaper article refers to the investigations of Mirza Musa Tabib-e Sāwji, a public health physician during the Qajar era, regarding sore throat, exanthematous eruptions, and profuse sweating in the axillae, groin, and lower abdomen, symptoms suggestive of makhmalek (likely typhoid fever), which reportedly affected Iranian children, particularly in Isfahan, over a four-year period. This clinical description aligns with the observations of the French physician and epidemiologist Dr. Joseph Désiré Tholozan (1820‒1897). According to the newspaper’s author, this disease had not been previously documented in Iran; however, it had been recognized in Europe for over a century by the French physician Pierre Fidèle Bretonneau (1778‒1862). (It should be noted that in historical sources, febrile illnesses such as scarlet fever and makhmalek (typhoid fever) were sometimes conflated due to the difficulty in clinically distinguishing between them prior to the 20th century.)

 Mehdi Qoli Khan Hedayat Mokhber ol-Saltaneh (1863–1955) was a prominent Iranian intellectual, writer, and statesman serving as Minister of Science, governor of Azerbaijan and Fars, and known for his literary and rhetorical talents. He recalled falling ill with scarlet fever at the age of 23, an episode during which Dr. Isidor Albou, a European physician and medical instructor at Dar ul-Funun, visited him at home to provide treatment. According to Mokhber-ol Saltaneh:”The illness had such an effect on me that in my weakest state, while unconscious, I spoke to my mother in German”.^38^

 In the book “Marat al-Waqi’at Mozaffari”, written by Abdul Hossein Khan Sepehr (1871–1933), better known as Malek al-Morkhin, the author chronicles events in Iran and its people from April 1897 to 1907, during the reign of Naser al-Din Shah’s successor, Mozaffar ad-Din Shah Qajar (r. 1896–1907). Regarding the outbreak of scarlet fever, he wrote: “This year (corresponding to September 1899) the heat in Tehran reached an unprecedented level, causing widespread illness among the population, especially among children, who were particularly affected by diseases like scarlet fever (*Mokhmalak*) and measles (*Sorkhcheh*)”.^39^

 Qahraman Mirza Salur (1872–1945) better known as Ayn al-Saltanah, a high-ranking official, provincial governor, and memoirist, mentioned in his writings the successful treatment of scarlet fever by an American physician in just two weeks, compared to the treatment duration of up to three months when managed by Iranian doctors. Based on this observation, he concluded that Iran needed hospitals modeled after the USA system.^40^

## Scarlet Fever and Iranian Physicians

 Research through the National Library and Archives of Iran in Tehran identified several 19th- and early 20th-century Persian medical texts referencing scarlet fever. Among them are Zinat al-Abdan and Shafa’iya by Dr. Johan Louis Schlimmer, revised and rendered into contemporary Persian medical terminology by Mohammad Taqi ibn Mohammad Hashem Hakim-bashi Ansari Kashani (d. 1886), an Isfahani physician, scholar, and calligrapher. These works were lithographed by the Dar al-Funun press in 1862. Shafa’iya, originally compiled at Dar al-Funun in March 1856, includes some of the earliest known Persian descriptions of scarlet fever in its first volume.^41^

**Figure 5:**
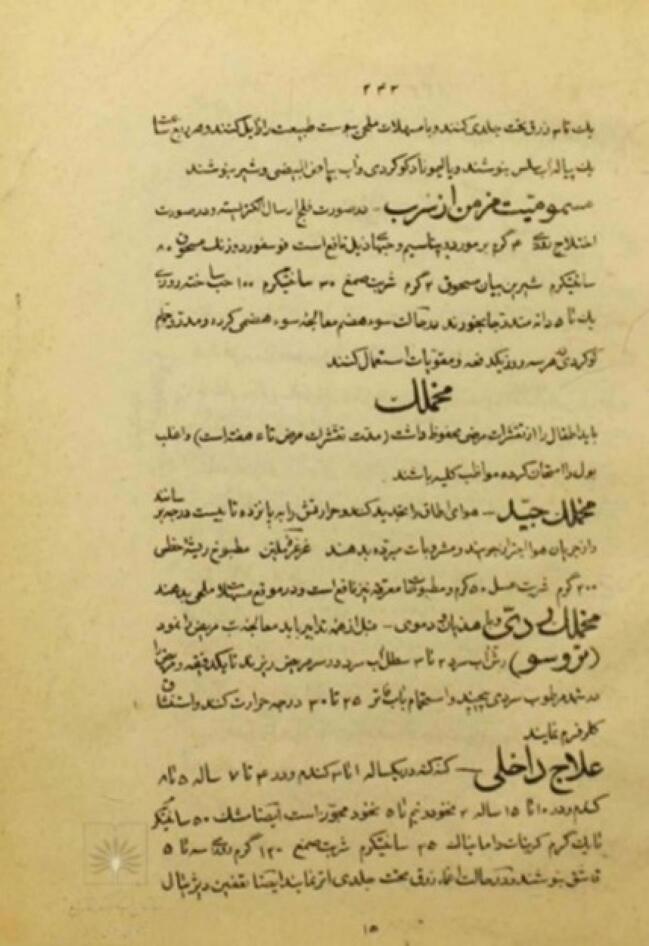
Treatment of Scarlet Fever in Khulasa al-Hikmeh by Zeyn al-Abedin ibn Shukrallah Qajar Ghavanloo and Mirza Mohammad Jazini Hamedani^41^

 Another significant source is Khulasat al-Hikmeh, a 19th-century treatise authored by Zayn al-‘Abedin ibn Shakrullah Qajar Ghavanloo and his student Mirza Mohammad Jazini Hamadani, who drew upon French medical literature. This text details therapeutic approaches to various illnesses, with specific instructions for treating scarlet fever on pages 242–243 (Figure 5).^41^

 Among these renowned scholars and physicians was Dr. Ali Khan ibn Zayn al-‘Abedin Hamadani, known as Mirza Ali Doctor, who lived during the reign of Naser al-Din Shah Qajar (1831–1896). He was born in Hamedan in 1845 and passed away in Tehran in 1892. He authored numerous works, one of which is “Ihya al-Atfal al-Nasiri (Mazfari)”. This book includes an introduction, two chapters, several sections, and a conclusion. Crucially, the text specifically mentions Scarlet Fever occurrence in children.^42,43^

**Figure 6:**
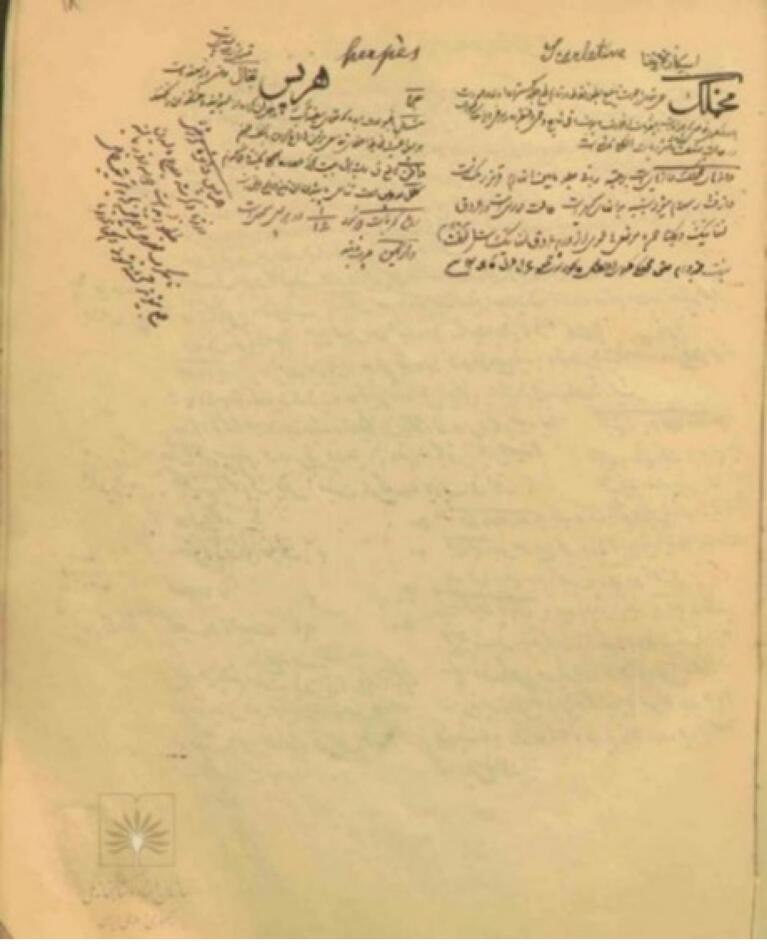
Description of Scarlet Fever in the manuscript “Amarāż-e Jildiyyah” (Skin Diseases), written by an unknown Persian author, likely from the 19th century^41^

 Two anonymous Persian manuscripts further document the disease. The first, Amarāż-e Jildiyyah (Skin Diseases), likely dates to the 19th century and is held at the National Library and Archives of Iran (retrieval no. 5-18382). Comprising 60 leaves (175 × 220 mm), it mentions scarlet fever on leaf 37.^41^ (Figure 6).

 The second, Amarāż-e Buthuriyyah (Eruptive Diseases), is preserved at the Mar’ashi Manuscript Center in Qom (catalog no. 18092/2). This 72-leaf manuscript discusses contagious diseases—including smallpox, measles, scarlet fever, and possibly chickenpox or typhoid. Though attributed to an anonymous author, internal evidence suggests it is a Persian adaptation of a European physician’s work, possibly transcribed by an Iranian student. It includes clinical case descriptions, one dated 1911 (leaf 66), and references the author’s teaching activities.^41^

 A fifth manuscript, Risālah-ye Pātulūjī-ye Dākhili (Treatise on Internal Pathology), is also anonymous and likely 19th-century. Housed at the National Library and Archives of Iran (retrieval no. 5-21741), it spans 83 leaves (115 × 183 mm). After a historical overview of medicine and a general pathology section, it notes on its final page that scarlet fever was a common illness in Iran.^41^

## Conclusion

 During the Qajar era, scarlet fever emerged as a clinically recognized yet inconsistently documented disease, occupying a complex space in Persian medicine. European physicians like Schlimmer and local practitioners identified its characteristic symptoms (fever, pharyngitis, and the telltale rash) though diagnostic ambiguities with measles or smallpox persisted. Traditional Iranian medicine notably distinguished between benign (*Mokhmalak)* and malignant (*Margejeh*) forms, reflecting nuanced observation. Outbreaks followed seasonal patterns, peaking in autumn and winter, as evidenced by reports from Isfahan (1873) and Tehran (1899), where extreme heat preceded pediatric epidemics. Urban centers and trade routes, including the Tabriz - Qazvin corridor, were particularly vulnerable, suggesting environmental and social transmission factors. Treatment approaches revealed stark disparities: mineral spring therapies and local remedies coexisted with European interventions, while elite accounts like Mokhber ol-Saltaneh’s memoir, detailing his delirium under Dr. Albou’s care, highlighted the disease’s severity. The 1899 Tehran outbreak, documented by Malek al-Morkhin, and Ayn al-Saltanah’s comparison of American versus Iranian treatment efficacy (two weeks versus three months) underscored both the disease’s toll and the Qajar medical landscape’s inequalities. Social stigma compounded clinical challenges, with some communities isolating patients like lepers. Despite its impact, scarlet fever’s historical footprint remains fragmentary. Limited mortality data, diagnostic ambiguities, and sparse newspaper coverage obscure its true burden, though surviving manuscripts like *Zinat al- Abdan, Shafa’iya*, *Khulasa al- Hikma*, *Amarāż -e Buthuriyyah* and *Risālah -ye Pātulūjī -ye Dākhili* confirm its persistent presence. This evidence collectively illustrates how Qajar Iran negotiated infectious disease within evolving local and global medical paradigms — a dynamic still relevant to modern public health historiography. Further research could illuminate transmission patterns through archival comparisons or spatial analysis of outbreak records.
